# Effects of follicular and luteal cysts on reproductive organs, interstitial glands, and mast cell distribution in buffalo ovaries

**DOI:** 10.1038/s41598-025-94941-y

**Published:** 2025-04-14

**Authors:** Yahia A. Amin, Amna H. M. Nour, Ragab H. Mohamed

**Affiliations:** 1 Department of Theriogenology, Faculty of Veterinary Medicine, Aswan University, Aswan, 81528 Egypt; 2 Department of Zoology, Faculty of Science, Aswan University, Aswan, Egypt

**Keywords:** Follicular cyst, Luteal cyst, Interstitial glands, Mast cell, Ovarian tissue, Oviduct, Uterus, Cell biology, Developmental biology, Systems biology, Zoology

## Abstract

A significant factor contributing to reproductive failure in dairy cattle that raised the possibility of culling was ovarian cysts. Its etiology and pathogenesis remained a puzzle, but investigation of the associated tissue modulation, particularly those of the ovaries, oviduct, and uterus, might shed some light on its development. Therefore, the current study aimed to assess changes induced by follicular and luteal cyst formation in the ovary, oviduct, and uterus. In addition, the aims also involved the effect of these cyst formations on the interstitial glands and mast cell distribution in the ovaries of dairy cows. Genital organs of healthy, non-pregnant buffalo-cows (*n* = 45) were collected from the abattoir. According to the ovarian status, buffalo-cows were divided into three equal groups (15 for each): one normal healthy group was the control group (Ctrl group) and two diseased groups. The first diseased group was the group of buffalo-cows with follicular cysts (FC group), while the second one was the group with luteal cysts (LC group). Blood and tissue samples were collected to determine progesterone levels and do histological investigations of the reproductive organs. Hematoxylin and eosin, Alcian blue-PAS, and Alcian blue-safranin-O stains were used for investigating ovarian tissues, interstitial glands (IGs), and mast cells (MCs), respectively. Results showed that significantly reduced thickness of the ovarian cortex and medulla, loss of ovarian folliculi, hemorrhage, and dilated blood vessels were observed in the FC and LC groups compared to the Ctrl group. In addition, the cystic ovaries significantly reduced interstitial gland count that was characterized by histopathological alterations that included atrophied and apoptotic cells and fragmentation, fading, and pyknotic nuclei. Likewise, in cystic ovaries, mast cell counts were found significantly reduced compared to the Ctrl group. The ovarian cysts significantly reduced the length and diameter of oviductal mucosal villi that were characterized by severe histopathological fluctuation in the ciliated cells and protruding and non-protruding secretory cells. For the uterus, the average thickness of the myometrium and endometrium in the ovarian cyst groups was significantly reduced compared to the Ctrl group. Furthermore, histopathological changes in the uterine glands, including severe apoptotic alterations, fading, pyknotic, and fragmented nuclei, were observed. In conclusion, the current study indicated that follicular and luteal cyst formations in the ovary induced various changes in the reproductive organs, interstitial glands, and mast cell distribution in the ovarian stroma, providing insights into the potential pathogenesis of cyst formation.

## Introduction

The postpartum period played a pivotal role in cattle reproduction. This period was considered critically important due to the common complications associated with various reproductive problems, including retention of the placenta^1^, endometritis^2,3^, opportunistic bacterial invasion^4^, inactive ovaries^5–7^, pyometra and ovarian cyst^8^.

During the postpartum period, ovarian cysts were one of the major factors contributing to dairy cattle’s inability to reproduce. The high occurrence of ovarian cysts often resulted in significant financial losses, longer waiting times for the first service, increased open days, and a higher risk of culling^8^.

The incidence of cystic ovarian follicle (COF) formation ranged from 6 to 30%^9,10^. Compared to heifers or beef cows, multiparous, lactating dairy cows were more frequently affected by COF^11^. COFs were typically characterized as anovulatory follicles larger than 25 mm in diameter, developing on one or both ovaries, and persisting for more than 10 days without luteal tissue^12^. However, some definitions indicated that the cysts were smaller, ranging from 17 to 20 mm in diameter, and lasted for a shorter duration^13,14^. The majority of research categorized cystic ovarian follicles (COFs) as luteal or follicle-theca cysts based on their hormonal composition and macroscopic characteristics. Follicle cysts were typically thin-walled and released estrogen, while luteal cysts, which had walls thicker than 3 mm, produced high levels of progesterone^14^. COFs were dynamic structures that could either form new cysts, persist, regress spontaneously, or transition from follicular to luteal cysts^11,12^.

Previous studies identified the theca interna (TI) of atretic follicles as the origin of a structure with an epithelial-like appearance, referred to as interstitial glands^15^. In species such as rabbits and rats, atretic processes transform thecal cells into interstitial cells that secrete hormones. It has long been recognized that this tissue was more prevalent during estrus, with progesterone and interstitial gland cells appearing to increase during pregnancy^16^.

It remained unclear what role the interstitial gland played in the ovarian physiology of mammals. The interstitial gland might be considered a species-specific endocrine steroid-producing structure in the ovary^17^ with the ability to synthesize and store steroid hormones^18^. In humans, the interstitial glands secrete estrogen, while the well-developed glands in rabbits secrete progesterone. Until now, little has been known about this structure and its role in the physiological and pathological conditions of the ovary.

Although the exact cause of cystic ovarian follicles (COF) remained unknown, metabolic, immunological, and hormonal abnormalities wer found to contribute to the initiation of cystic ovarian formation^19,20^. One immunological aspect related to ovarian diseases was the distribution of mast cells, which were known to exhibit different morphological and functional characteristics depending on their location within the organism^21^. Numerous investigations demonstrated that the distribution of mast cells (MCs) in the female reproductive organs varied depending on the stage of the estrous cycle in species such as bitches^22^, sows^23,24^, ewes^25^, goats^26^, cows^27^, and mares^28,29^. Additionally, it was proposed that, in certain species, MCs were involved in embryo implantation in the uterus^30,31^. The lack of understanding regarding mast cell localization and distribution in female reproductive organs highlighted the importance of investigating these immune cells.

The current study hypothesizes that the mechanism of cyst formation is associated with variable changes in ovarian tissue and other reproductive organs, such as the oviduct and uterus. In addition, IGs and MCs distribution may also have a role in this mechanism. Therefore, this study aimed to assess the variant changes induced by follicular and luteal cyst affections on the ovarian tissue, oviduct and uterus. In addition, the aims also involved the effect of these cyst formations on the IGs and MCs distribution in the ovarian tissue of the buffalo cows.

## Materials and methods

### Animals and sample collection

All procedures were carried out according to the guide that was approved by the Ethics Committee of the Faculty of Veterinary Medicine, South Valley University, Egypt (approval number VM/SVU/24(9)-02). All methods were performed in accordance with the relevant guidelines and regulations. Forty-five female buffalo-cows, aged five to seven that were slaughtered at the Daraw slaughterhouse in Aswan province and appeared to be in a good health were included in the study. The genital tracts were removed after slaughter, put in separate containers with ice bags (4° C), and delivered to the lab in less than an hour. There were no genital illnesses or disorders present in the gathered genital tracts. Ultrasonographic exams were performed on each ovary using an ultrasound device (Eickemeyer Magic 500 Digital, Germany) combined with a linear probe (5.0–7.5 MHz).

According to the ovarian status, buffalo-cows were divided into three groups with an equal number of animals in each group: normal healthy buffalo-cows that were considered the control normal group (Ctrl group). The second group consisted of fifteen diseased buffalo-cows that suffered from follicular cyst formation on the ovaries and was considered the follicular cyst (FC) group. The third group consisted of fifteen diseased buffalo-cows that suffered from luteal cyst formation on the ovaries and was considered the luteal cyst (LC) group. Differentiation of the groups depends on the ovarian findings as the following: the control normal group did not have cyst formation on the ovary. The follicular cyst (FC) group revealed the presence of the follicular cyst that was characterized by a size of 3–5 cm, a thin wall ≤ 0.3 cm, and progesterone concentration < 1 ng/mL. Luteal cyst (LC) group characterized by presence of the lutein cyst that was typified by a diameter of 3–5 cm, a cavity filled with liquid, a thick luteinized wall with a thickness > 0.3 cm and progesterone concentration > 1 ng/mL^32,33^.

### Blood sampling and hormonal analysis

Blood samples were collected from the jugular vein prior to slaughter. After allowing the samples to clot, they were centrifuged for 15 min at 4 °C at 2500 rpm. Serum samples were then transferred to 1.5 mL Eppendorf tubes and stored at -20 °C. The progesterone concentration was determined using the direct ELISA technique (Human PROG ELISA, GmBH, Germany). The assay sensitivity was 0.06 ng/mL, with inter-run and intra-run coefficients of variation of 4.5% and 2.6%, respectively.

### Histological and histochemical staining

Sections of the ovary, oviduct, and uterus were excised, cleaned in sterile saline, and preserved in 10% neutral phosphate-buffered formalin (pH 7.0). For microscopic examination, tissue sections of 5 μm thickness were prepared and stained with Harris hematoxylin and eosin (a standard stain for histological investigation), Alcian blue-PAS stain to highlight interstitial glands, and Alcian blue-safranin-O stain to visualize mast cells^34^. Specimens were dehydrated in a series of ethyl alcohol solutions (50–99%), cleared in methyl benzoate, and embedded in molten paraffin wax at 58–62 °C. High-power light microscopy (Olympus BX43F, Tokyo, Japan) was used to examine the stained sections, and image analysis was performed using a personal computer and camera software (Olympus DP74, Tokyo, Japan). The cell count in the images was conducted using Image J software.

### Immunohistochemecal staining

Tryptase, a protease stored in the secretory granules of mast cells, immunohistochemistry staining was used to identify and quantify mast cells in ovarian tissue, providing insights into their distribution and potential role in the pathophysiology of follicular and luteal cysts. Graded alcohols were used to hydrate 5 μm ovarian sections after they had been deparaffinized in xylene. The sections were then cooked in citrate buffer at pH 6 for 10 min in a microwave oven. 3% H_2_O_2_ in 100% methanol was used to suppress endogenous peroxidase activity for 15 min at room temperature, followed by serum block with 10% goat serum (ab 7481, Abcam Co., UK) in phosphate buffer saline (PBS) for one hour at room temperature, then rinse in PBS. The sections were incubated with mouse primary tryptase antibody (ab2378, Abcam co. UK) in PBS with 1% goat serum and 0.1% triton at (1/100) for an overnight period at 4 °C, and then rinse 3x in PBS. (1:100) diluted HRP-conjugated anti-rabbit secondary antibody (#31460, Thermo fisher Co., USA) was added and then rinse 3x in PBS, followed by applying diaminobenzidine for one minute then rinse in distilled water, hematoxylin staining was done for one minute followed by rinsing in tap water for 10 min. After that, dehydrated through graded alcohols to xyleen. DPX was used to mount the specimens.

### Statistical analysis

Results of all quantitative data of the histological analysis were expressed as means ± SD. Levene’s test for homogeneity of variance and the Anderson-Darling normality test were performed. Differences between means were tested by one-way analysis of variance ANOVA followed by the Student-Newman-Keuls T-test using Minitab 12 software so that the data obtained can be compared and statistically evaluated.

## Results

**Fig. 1:**
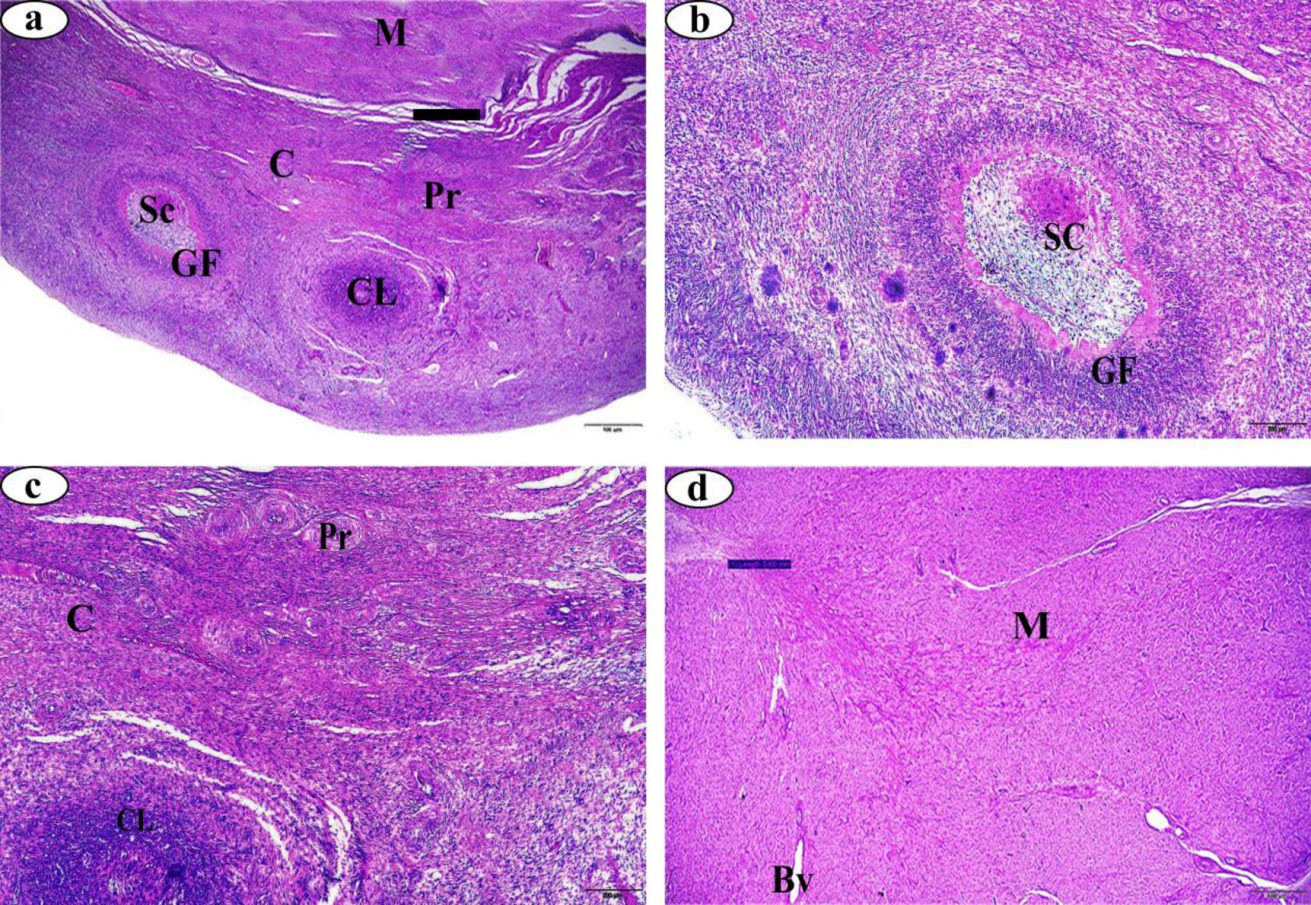
Photomicrographs of normal ovary stained with H&E. (**a**) cortex section 40X magnification, (**b** &** c**) cortex section 100X magnification (**d**) medulla section 40X magnification. Cortex (*C*), medulla (*M*), primary follicle (*Pr*), secondary follicle (*Sc*), Graafian follicle (*GF*), corpus luteum (*CL*), blood vessel (*Bv*).

**Fig. 2:**
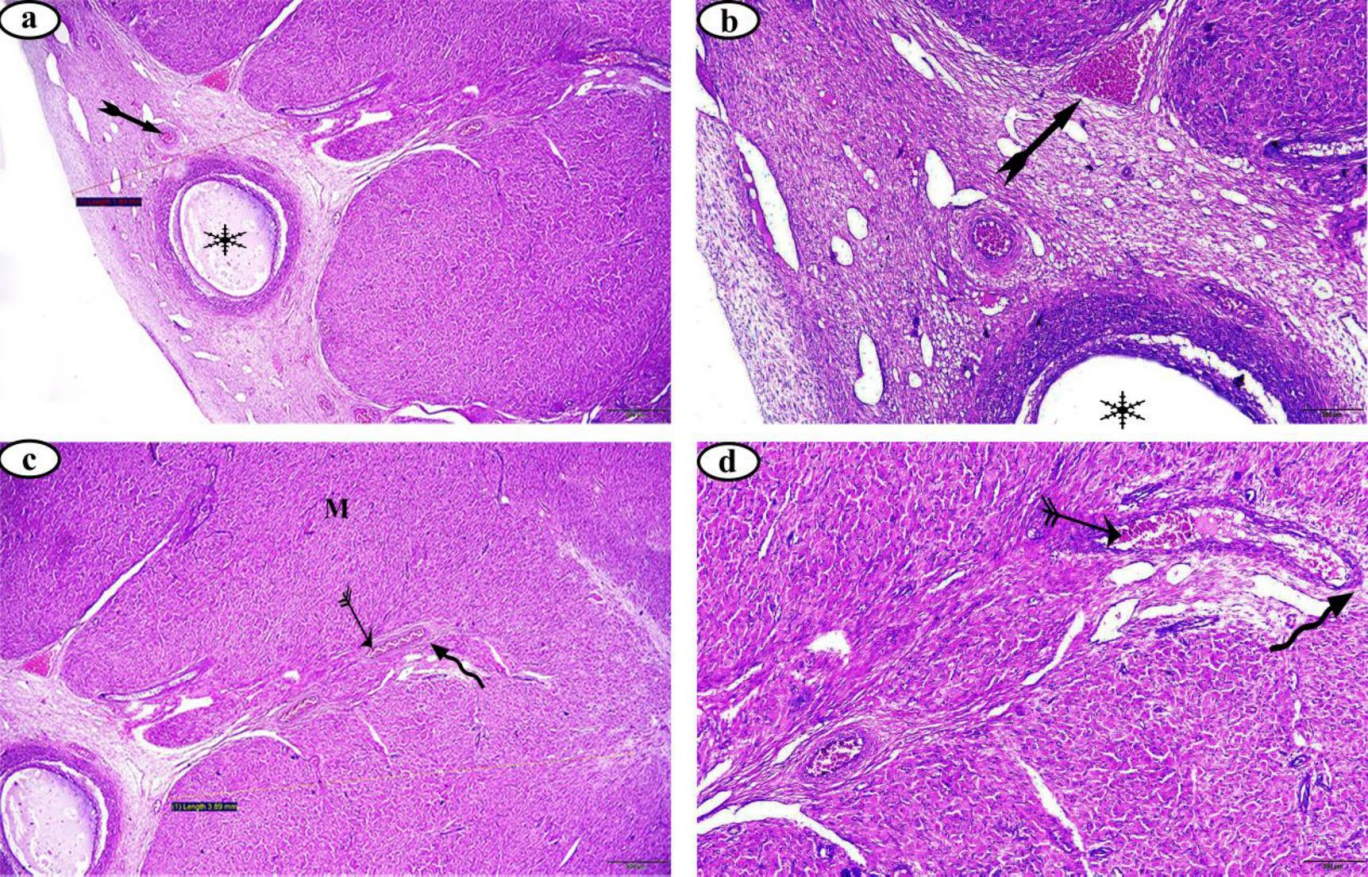
Photomicrographs of follicular cysts of ovary stained with H&E. (**a**) cortex section 40X magnification, (**b**) cortex section 100X magnification (**c**) medulla section 40X magnification, (**d**) medulla section 100X magnification. medulla (*M*), hemorrhaged blood vessel  , dilated blood vessel  , apoptotic oocytes , and fibrotic C. T.  .

**Fig. 3:**
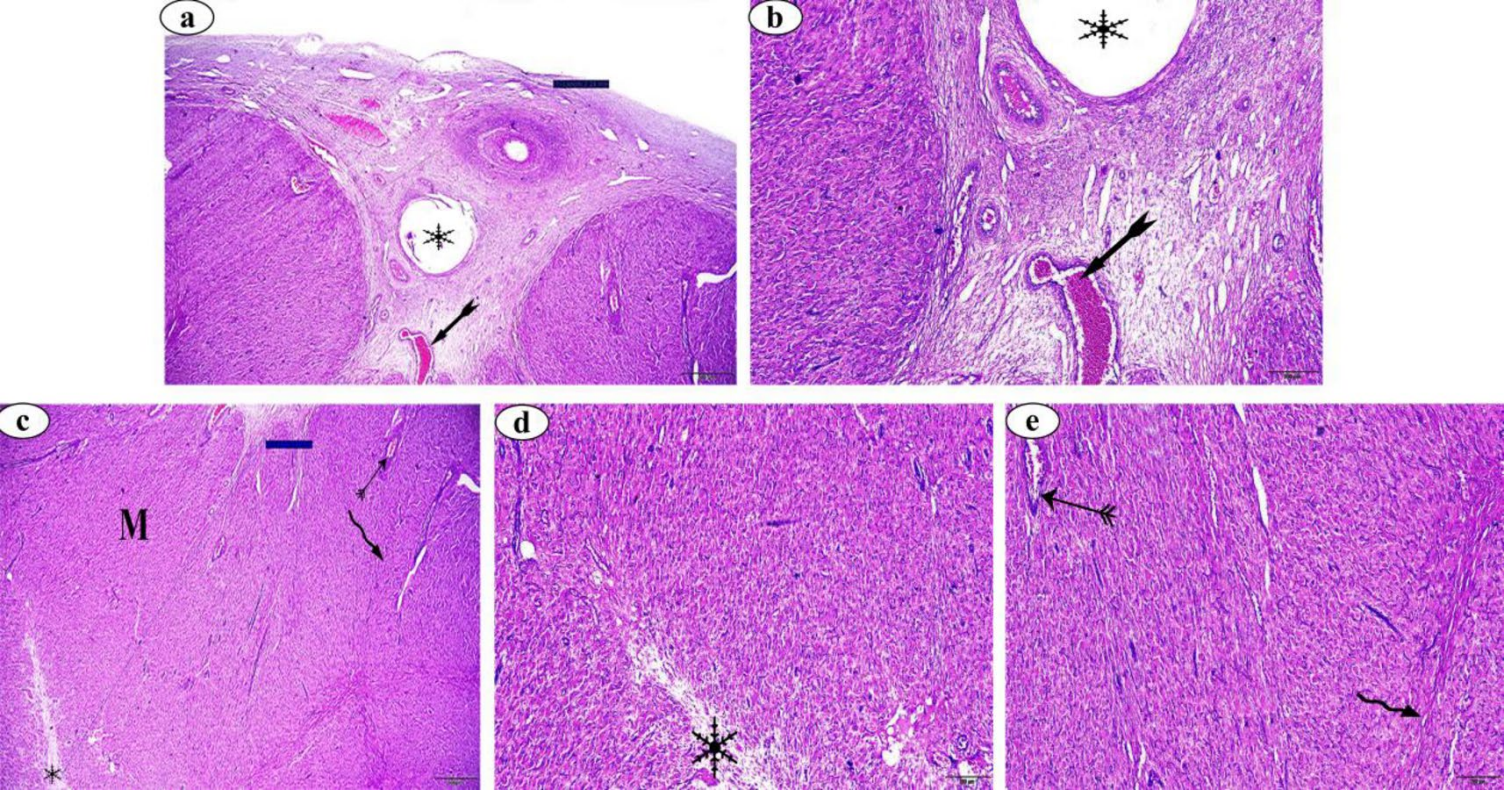
Photomicrographs of luteal cysts of ovary stained with H&E. (**a**) cortex section 40X magnification, (**b**) cortex section 100X magnification (**c**) medulla section 40X magnification, (**d** &** e**) medulla section 100X magnification. medulla (*M*), hemorrhaged blood vessel  , dilated blood vessel  , apoptotic oocytes , and fibrotic C. T.  .

Results of the P4 hormone assessment revealed that buffalo-cows of the FC group had progesterone levels < 1 ng/ml, while that of the LC group had progesterone levels > 1 ng/mL. The current study also investigated the histological changes within ovarian cortex, and medulla, oviduct mucosa, myometrium and endometrium. Results showed that the average thickness of the ovarian cortex was 3.79 ± 0.67 mm in the Ctrl group, 2.02 ± 0.57 mm in the FC group, and 1.81 ± 0.25 mm in the LC group. Similarly, the average thickness of the ovarian medulla was 5.61 ± 0.96 mm in the Ctrl group, 3.80 ± 1.05 mm in the FC group, and 3.63 ± 1.06 mm in the LC group (Table 1). There was a significant decrease in the average thickness of the ovarian cortex and medulla of the FC and LC groups compared to normal Ctrl group (*p* > 0.05 and 0.01).Furthermore, contrary to the Ctrl group (Fig. 1),  results showed the presence of numerous histopathological changes in the ovarian tissue, such as apoptotic oocytes of all generations, fibrotic connective tissue, hemorrhage and dilated blood vessels in the FC (Fig. 2) and LC (Fig. 3) groups.

**Table 1:** Mean ± S.D of ovarian cortex and ovarian medulla of the ovary of control group (*Ctrl*), follicular cyst group (*FC*) and luteal cyst group (*LC*).

	Ctrl group	FC group	LC group
Ovarian cortex thickness	3.79 ± 0.67 mm	2.02 ± 0.57 mm *	1.81 ± 0.25 mm *
Ovarian medulla thickness	5.61 ± 0.96 mm	3.80 ± 1.05 mm **	3.63 ± 1.06 mm **

(*) Significant compared to a normal ovary (*p*<0.05).

(**) Highly significant compared to a normal ovary (*p*<0.01).

**Fig. 4:**
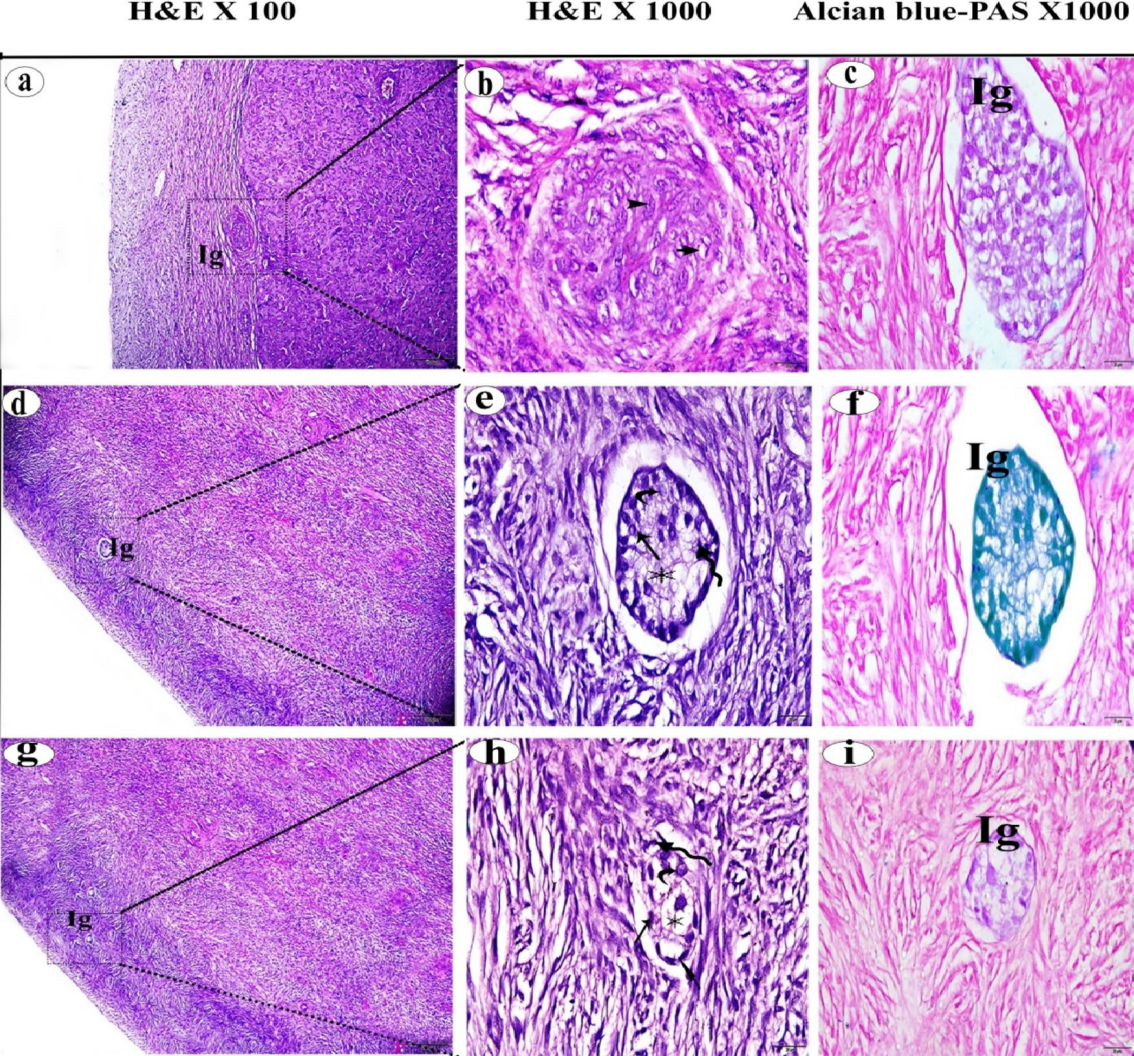
(**a**–**c**) Photomicrographs of normal interstitial glands stained with H&E and PAS-Alcian blue, respectively. (**d**–**f**) Photomicrographs of interstitial glands of follicular cystic ovary stained with H&E and PAS-Alcian blue, respectively. (**g**–**i**) Photomicrographs of interstitial glands of luteal cystic ovary stained with H&E and PAS-Alcian blue, respectively. Interstitial gland (*Ig*), epithelial cells  , adipocytes , pyknotic nuclei  , nuclear fragmentation  , nuclear fading (arrow) and apoptotic cells  .

In the control group, the interstitial glands (Fig. 4a), lined by epithelial cells (Fig. 4b), varied in shape from cuboidal to polyhedral. These glands had large, oval nuclei and clear cytoplasm due to the accumulation of lipid droplets (Fig. 4b,c). In contrast, the interstitial glands in the FC (Fig. 4d,e,f) and LC (Fig. 4g,h,i) groups exhibited histopathological alterations, including apoptotic cells with fragmented, faded, and pyknotic nuclei, which were not observed in the Ctrl group.

The average ovarian interstitial cell count for the three tested groups was presented in Table 2. The number of mast cells (MCs) was 6.72 ± 1.03 in the normal Ctrl group, 3.92 ± 0.83 in the FC group, and 3.02 ± 0.27 in the LC group. A significant decrease in the number of MCs was observed in both the FC and LC groups compared to the normal Ctrl group (*p* < 0.01).

**Table 2:** Mean ± S.D of ovarian interstitial gland (gland / mm3) and mast cells (cell / mm3) count of control group (*Ctrl*), follicular cyst group (*FC*) and luteal cyst group (*LC*).

	Ctrl group	FC group	LC group
Ovarian interstitial cells count	6.72 ± 1.03	3.92 ± 0.83 *	3.02 ± 0.27 *
Ovarian mast cells count	14.82 ± 2.87	3.57 ± 0.73 **	2.57 ± 0.67 **

(*) Significant compared to a normal ovary (*p*<0.05).

(**) Highly significant comparing to normal ovary (*p*<0.01).

**Fig. 5:**
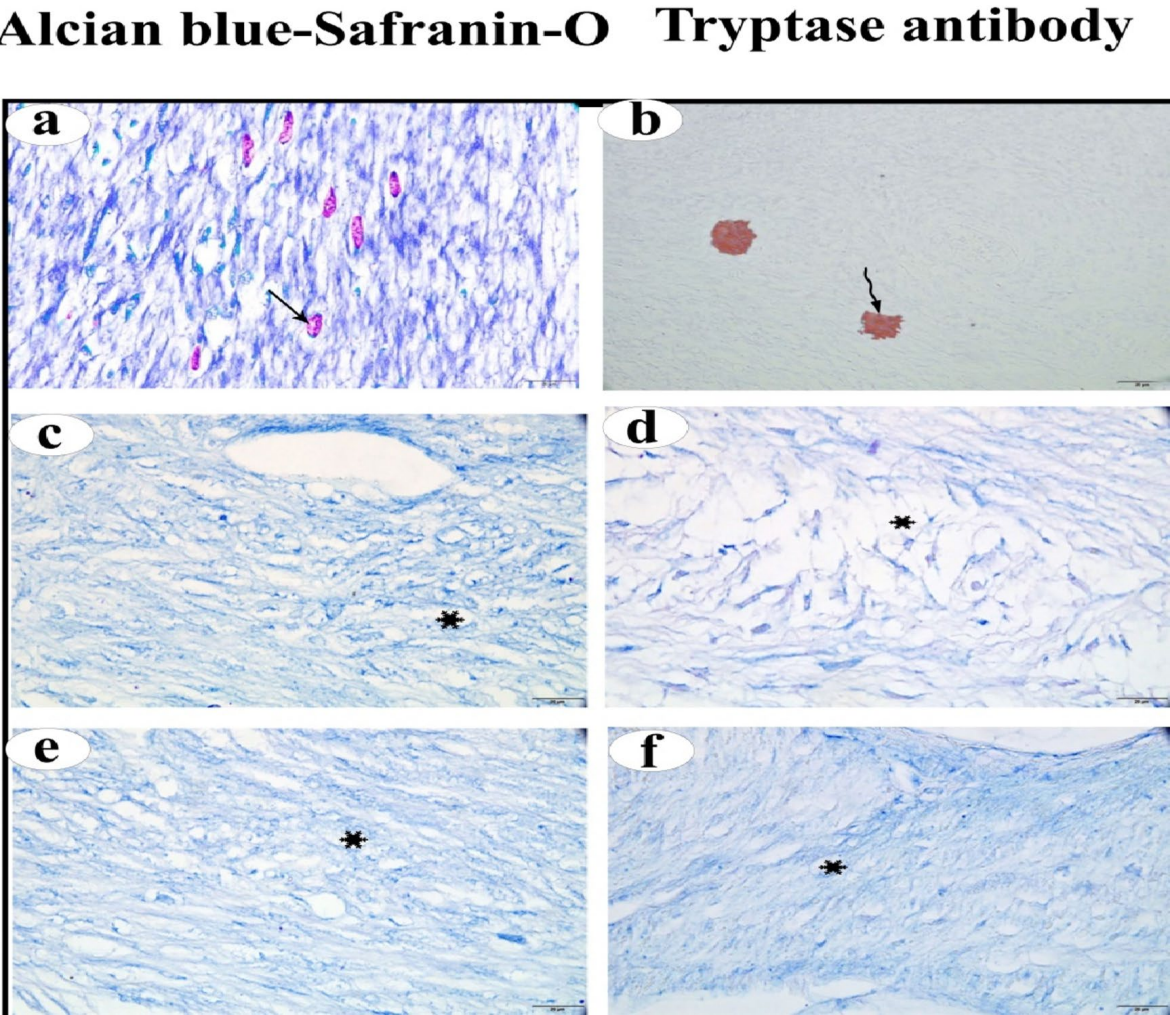
(**a**,** b**) Photomicrographs of normal ovarian mast cells stained with Alcian blue- Safranin-O and Tryptase immunohistochemical stain, respectively. (**c**,** d**) Photomicrographs of mast cells of follicular cystic ovary stained with Alcian blue- Safranin-O and Tryptase immunohistochemical stain, respectively. (**e**,** f**) Photomicrographs of ovarian mast cells of luteal cystic ovary stained with Alcian blue- Safranin-O and Tryptase immunohistochemical stain, respectively. Mast cells (arrow), tryptase immunopositive granules  , mast cell count reduction  .

The average mast cell counts in the ovarian cortex were shown in Table 2. The number of MCs was 14.82 ± 2.87 in the normal Ctrl group, 3.57 ± 0.73 in the FC group, and 2.57 ± 0.67 in the LC group. A significant decrease in the number of MCs was observed in both the FC and LC groups, as indicated by the negative Alcian Blue Safranin-O stain and the negative expression of tryptase, compared to the normal Ctrl group (*p* < 0.01) (Fig. 5).

The average length and diameter of the oviduct mucosal villi in the three tested groups were presented in Table 3. The average length of the oviduct mucosal villi was 636.56 ± 105.78 μm in the normal Ctrl group, 164.63 ± 124.86 μm in the FC group, and 123.61 ± 32.87 μm in the LC group. The average diameter of the oviduct mucosal villi was 53.68 ± 16.86 μm in the normal Ctrl group, 64.50 ± 21.86 μm in the FC group, and 62.35 ± 17.97 μm in the LC group.

**Table 3:** Mean ± S.D of oviduct mucosa, myometrium, and endometrium measurements of control group (*Ctrl*), follicular cyst group (*FC*) and luteal cyst group (*LC*).

	Ctrl group	FC group	LC group
Oviduct mucosal villi length	636.56 ± 105.78 μm	164.63 ± 124.86 μm **	123.61 ± 32.87 μm **
Oviduct mucosal villi diameter	53.68 ± 16.86 μm	64.50 ± 21.86 μm *	62.35 ± 17.97 μm *
Myometrium thickness	3.48 ± 1.07 mm	1.89 ± 0.57 mm *	1.53 ± 0.56 mm *
Endometrium thickness	6.95 ± 1.57 mm	4.84 ± 1.57 mm *	3.64 ± 1.17 mm *

(*) Significant compared to a normal ovary (*p*<0.05).

(**) Highly significant compared to a normal ovary (*p*<0.01).

Significant differences were observed in both the length and diameter of the oviduct mucosal villi between the three tested groups. A significant decrease in the length of the oviduct mucosal villi was observed in the FC and LC groups compared to the normal Ctrl group (*p* < 0.01). In contrast, the diameter of the oviduct mucosal villi in the FC and LC groups showed a significant increase compared to the normal Ctrl group (*p* < 0.05).

**Fig. 6:**
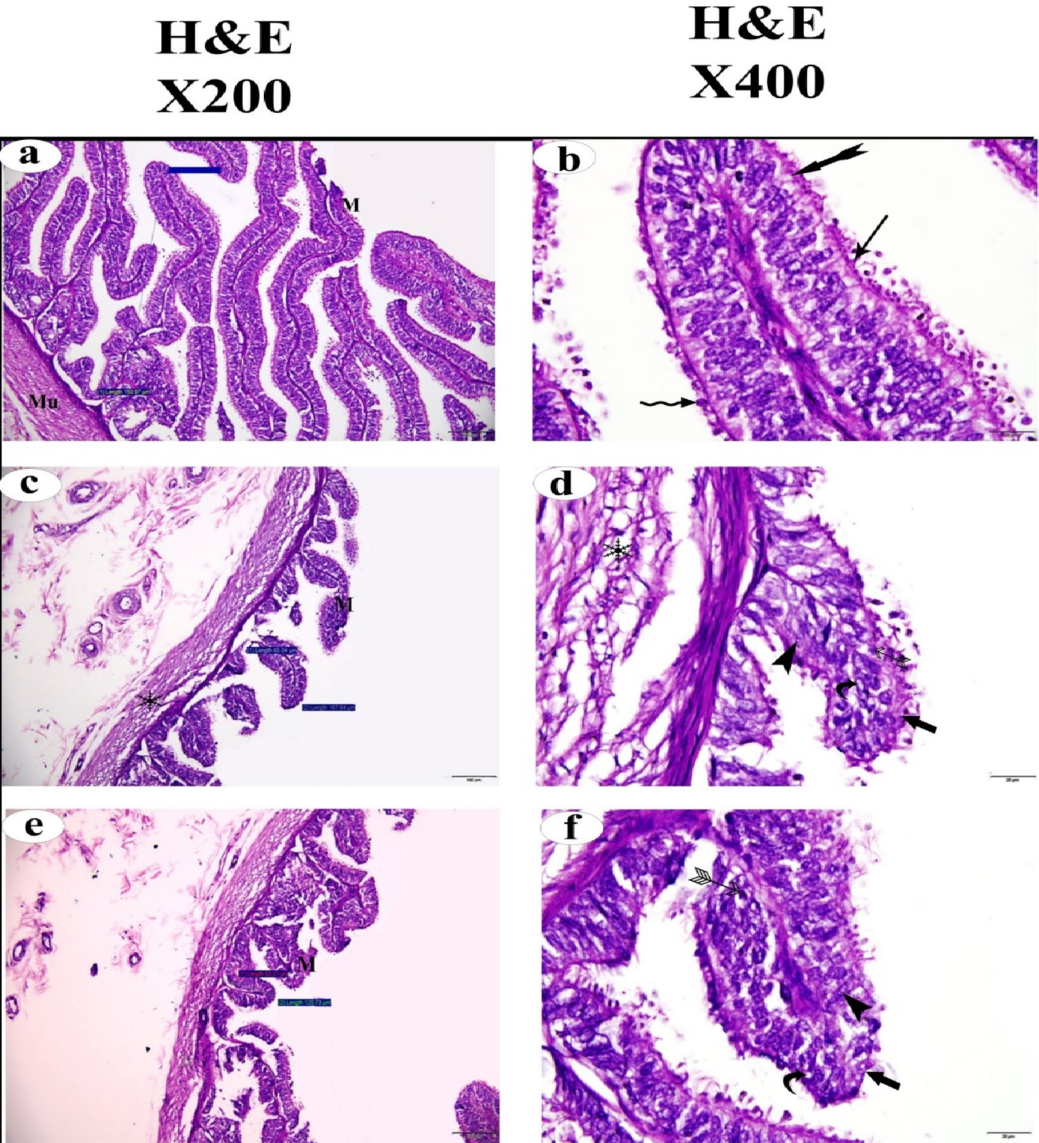
(**a**,** b**) Photomicrographs of normal oviduct stained with H&E. (**c**,** d**) Photomicrographs of oviduct of follicular cystic ovary stained with with H&E. (**e**, **f**) Photomicrographs of oviduct of luteal cystic ovary stained with H&E. oviduct mucosa (*M*), muscularis (*Mu*), fragmented mascularis  , pyknotic nuclei  , nuclear fragmentation  , nuclear fading  and distorted mucosa (thick arrow).

Regarding the oviduct, H&E staining and histomorphological quantitative analyses were performed to assess the effect of ovarian cysts on the normal appearance of different epithelial cell types. The FC and LC groups showed severe histopathological alterations in the ciliated cells, non-protruding secretory cells, and protruding secretory cells. These changes were represented by a distorted mucosa with nuclear fragmentation, nuclear fading, pyknotic nuclei, and a fragmented muscularis layer (Fig. 6).

**Fig. 7:**
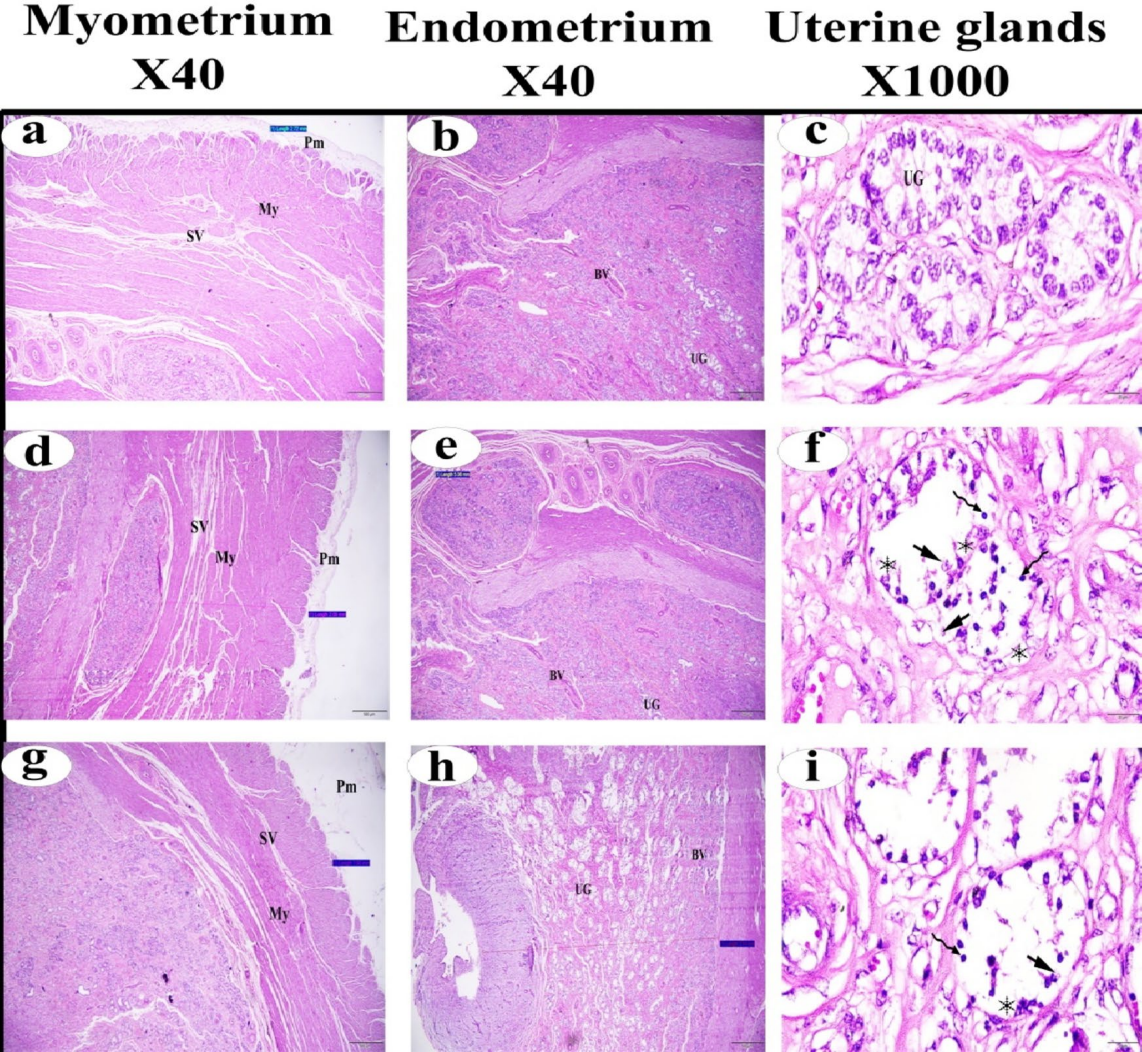
(**a**–**c**) Photomicrographs of normal uterus stained with H&E. (**d**–**f**) Photomicrographs of uterus of follicular cystic ovary stained with with H&E. (**g**–**i**) Photomicrographs of uterus of luteal cystic ovary stained with H&E. premetrium (*Pm*), myometrium (*My*), stratum vasculare (*SV*), uterine gland (*UG*), blood vessel (*BV*), apoptotic cells  , pyknotic nuclei  , and nuclear fragmentation  .

The average thickness of the myometrium and endometrium in the uterus for the three tested groups was shown in Table 3. The average thickness of the myometrium was 3.48 ± 1.07 mm in the normal Ctrl group, 1.89 ± 0.57 mm in the FC group, and 1.53 ± 0.56 mm in the LC group. The average thickness of the endometrium was 6.95 ± 1.57 mm in the normal Ctrl group, 4.84 ± 1.57 mm in the FC group, and 3.64 ± 1.17 mm in the LC group. A significant decrease in the thickness of both the myometrium and endometrium was observed in the FC and LC groups compared to the normal Ctrl group (*p* < 0.05). Histological examination revealed histopathological alterations in the uterine glands of the FC and LC groups compared to the normal Ctrl group. These alterations included severe apoptotic changes, fading, pyknotic nuclei, and fragmented nuclei (Fig. 7).

## Discussion

It is well established that the detection of progesterone concentration was used as an indicator for determining the degree of luteinization^35^. In the present study, the results of the progesterone (P4) assessment showed that buffalo cows in the FC group had P4 levels < 1 ng/mL, while those in the LC group had P4 levels > 1 ng/mL. Similar findings were reported in previous studies, which indicated that follicular cysts were characterized by low progesterone concentrations (< 1 ng/mL), whereas luteal cysts exhibited high progesterone levels^36^ .

The reproductive system was significantly impacted both morphologically and histologically by cystic ovaries, which could have contributed to the persistent infertility observed. In the present study, cyst formation resulted in thinning of both the cortex and medulla of the ovary. Furthermore, one of the most notable changes observed in the ovarian tissue of both the FC and LC groups was the dilation of blood vessels, which, in some cases, progressed to hemorrhage. However, limited research has investigated the morphological and histological alterations that occur in the ovary during cyst formation, as well as the effects of these changes on the ovary and its structures. Therefore, this study is the first to focus on these specific alterations. The findings of our study are consistent with those previously reported regarding the complications associated with cystic ovarian disease. While similar complications have been observed in humans, it has been suggested that these cases may lead to significant blood loss, resulting in hemodynamic instability^37^. Additionally, a study in local Baldi cows suffering from follicular cysts indicated that the primary pathological lesion in the ovarian medulla was congestion and hyalinosis of blood vessels, accompanied by mononuclear cell infiltration in some animals^38^.

The epithelial cells lining the IGs in this study ranged in shape from cuboidal to polyhedral, with large, oval nuclei and transparent cytoplasm due to the accumulation of lipid droplets. These findings were consistent with previous research conducted on camels, where the interstitial glands in the ovary were located in the cortical region and exhibited various arrangements, including single, paired, or grouped patterns^39^.

In the present study, the count of IGs was significantly decreased in the FC and LC groups compared to the normal Ctrl group. A previous study in buffaloes investigating ovarian IGs suggested that interstitial tissue could originate from various sources, but in mature female buffaloes, it predominantly developed from differentiated stromal cells and theca interna of atretic follicles^40^. Additionally, it has long been established that interstitial gland cells undergo hypertrophy and that progesterone levels are elevated during pregnancy. This tissue was found to be abundant during estrus and appeared to increase during pregnancy^16^. The induction of follicular and luteal ovarian cysts, which originated from non-ovulated follicles, could provide an explanation for the decreased IG count observed in the FC and LC groups in the present study.

The precise function of IGs in mammalian ovarian physiology remains unclear. It is suggested that IGs may synthesize and store steroid hormones and could be considered a species-specific endocrine steroid-producing structure in the ovary^17,18^. In humans, interstitial glands secrete estrogen, while progesterone is the primary hormone secreted by these glands in rabbits. Additionally, it has been confirmed that progesterone can be metabolized by the primary and secondary interstitial glands in rabbits^41^.

Prior research indicated that, following the onset of follicular atresia at three months of age, secondary IGs in adult rabbits originated from the theca interna (TI)^42^. The TI cells, located near the stromal or theca externa, developed into secondary IGs. Furthermore, the researchers found that early in the development of follicular atresia, TI cells of atretic follicles did not immediately transform into secondary interstitial glandular cells. However, in the later stages of atresia, the TI cells underwent cytoplasmic hypertrophy, and the granular reticulum appeared. Secondary interstitial cells were subsequently formed from the TI, a finding consistent with observations in guinea pigs^43^.

To the best of our knowledge, there is no documented information regarding the relationship between ovarian architecture, reproductive organs, and the distribution of MCs in the ovarian tissues of various animal species. Mast cells are considered to be novel mediators of reproductive processes.

In the current study, mast cells (MCs) showed a significant increase in their presence in the ovarian cortex of the control group, compared to their absence in the follicular cyst (FC) and luteal cyst (LC) groups. Previous research has reported that MCs were present in both the cortical and medullary tissues of cow ovaries, which exhibited a long estrous cycle and well-developed interstitial cortical stroma^44^. Other studies have found that MCs were only present in the medulla of short-cycle estrous rats and guinea pigs^45,46^. Additionally, a small number of MCs were identified in the stromal tissue of the human endometrium^47,48^. Recent studies have also shown that the location of MCs in female reproductive organs varied depending on the stage of the estrous cycle in species such as bitches, sows, ewes, goats, and cows^27^.

To date, however, there has been a lack of research elucidating the function and characterization of ovarian MCs throughout follicular cycles and gestation in various animal species, highlighting the need to clarify the role of ovarian MCs. According to some studies, MCs may release histamine during the preovulatory phase, which increases vascular permeability^49^. In addition to histamine, active MCs have been shown to release metalloproteinases, proteases, and vascular endothelial growth factor (VEGF). Importantly, the histamine production by MCs has been reported to affect myometrial contractility, embryo implantation, and ovulation^50^. It is well established that histamine increases blood flow to the ovary and enhances capillary permeability. Therefore, it is logical that MCs would regulate reproductive functions, given that metalloproteinases and proteases degrade the extracellular matrix, while VEGF plays a key role in orchestrating neovascularization^51^.

The mature follicular stage (follicles measuring 1.8–2.0 cm), which corresponded to the male tolerance phase (estrous period) in live camels, was when the highest number of MCs were observed in the ovary. Previous studies using a mouse model have explored the relationship between the quantity of MCs and pregnancy outcomes^52^. Mast cells appeared to reach their highest levels during the receptive phase (estrous)^52^. This finding could help explain the decreased count of MCs in the current study in the follicular cyst (FC) and luteal cyst (LC) groups, which represented the anestrous stage.

In the oviduct, fertilization and the initial stages of embryogenesis occur, with the movement of the ovum and conceptus facilitated by the cilia of oviductal cells. A healthy environment is essential for the development of secretory cells that produce oviductal fluid, which supports the developing conceptus. Anomalies in the gamete transport process and failure of oviductal fluid production can lead to infertility. In the current study, the condition of cystic ovaries influenced the oviduct’s morphological and histological configuration. These findings align with a previous study, which reported that the presence of ovarian cysts was associated with negative alterations in the oviduct mucosa, including a decrease in ciliated cells^53^. These changes significantly impacted oviduct function, contributing to the adhesion and accumulation of tubal folds, as well as oviductal blockage. Such alterations likely hindered the migration of gametes, thus preventing fertilization.

The mammalian uterus undergoes significant changes in both morphology and physiology throughout different reproductive phases. Endometrial glands produce and secrete substances essential for the survival and development of the conceptus. Our research demonstrated that in cases of cystic ovaries, morphological alterations occurred in both the myometrium and endometrium. In the uterus of the ovarian cyst groups, a significant reduction in the thickness of both the myometrium and endometrium was observed, accompanied by severe degeneration of the uterine glands. The effects of cystic ovaries on the uterus were consistent with findings from previous studies, which suggested that the endometrium, whose cellular components and tissue growth are responsive to circulating hormones, might be intricately affected by the endocrinological disorders associated with cystic ovarian disease^54^.

## Conclusion

The current study indicated that follicular and luteal cyst formations on the ovary induced variant changes in the reproductive organs, interstitial glands, and mast cell distribution in the ovarian stroma which might serve to give some light about pathogenesis of cyst formation. In addition, the acquired data demonstrated a critical relationship between the histological alterations in the ovary, oviduct, and uterine mucosa, as well as their association with ovarian cysts and reproductive organ dysfunction. If this condition persisted for a long period, these organs might undergo permanent alterations.

## Data Availability

All data generated or analyzed during this study are included in this published article.

## References

[1] Amin, Y. A. & Hussein, H. A. Latest update on predictive indicators, risk factors and ‘omic’technologies research of retained placenta in dairy cattle–A review. *Reprod. Domest. Anim.* **57**, 687–700 (2022). 35332584 10.1111/rda.14115

[2] Amin, Y., Mahmoud, A., Aref, M., Seddek, A. & Younis, W. Treatment of endometritis caused by different types of bacteria by cefoperazone antibiotic and its effect on the days open and pregnancy rate in dairy cows (field study). *J. Anim. Health Prod.* **10**, 311–319 (2022).

[3] Amin, Y. A., Abdelaziz, S. G. & Said, A. H. Treatment of postpartum endometritis induced by multidrug-resistant bacterial infection in dairy cattle by green synthesized zinc oxide nanoparticles and in vivo evaluation of its broad spectrum antimicrobial activity in cow uteri. *Res. Vet. Sci.* **165**, 105074 (2023). 37948844 10.1016/j.rvsc.2023.105074

[4] Amin, Y. A. et al. Abortion associated with postpartum opportunistic bacterial invasion reduces fertility and induces disturbances of reproductive hormones, hematological profile, and oxidant/antioxidant profiles in dairy cows. *J. Adv. Vet. Anim. Res.* **10**, 654 (2023). 10.5455/javar.2023.j721 PMC10868696 38370890

[5] Amin, Y. A. et al. Treatment of inactive ovaries of Holstein dairy cows by epidural injection of GnRH analogue (receptal) and its impact on the reproductive hormones, oxidant/antioxidant profile and micro and macro-elements profile. *Animals* **13**, 653 (2023). 36830440 10.3390/ani13040653 PMC9951676

[6] Amin, Y. A., El-Naga, E. M. A., Noseer, E. A., Fouad, S. S. & Ali, R. A. Synchronization with controlled internal drug release (CIDR) and prostaglandin F2α (PGF2α) influences oxidant/antioxidant biomarkers and mineral profile in summer-stressed anoestrous buffalo (*Bubalus bubalis*). *Theriogenology* **134**, 34–41 (2019). 31129479 10.1016/j.theriogenology.2019.05.014

[7] Amin, Y. & Said, A. The addition of Chitosan to GnRH analog induces ovarian resumption and improves conception rates in buffaloes. *Trop. Anim. Sci. J.* **44**, 1–9 (2021).

[8] Yimer, N., Haron, A. W. & Yusoff, R. Determination of ovarian cysts in cattle with poor reproductive performance using ultrasound and plasma progesterone profile. *Vet. Med. Open J.* **3**, 1–9 (2018).

[9] Vanholder, T. et al. Hormonal and metabolic profiles of high-yielding dairy cows prior to ovarian cyst formation or first ovulation post partum. *Reprod. Domest. Anim.* **40**, 460–467 (2005). 16149953 10.1111/j.1439-0531.2005.00601.x

[10] Kim, K. D., Ki, K. S., Kang, H. G. & Kim, I. H. Risk factors and the economic impact of ovarian cysts on reproductive performance of dairy cows in Korea. *J. Reprod. Dev.* **51**, 491–498 (2005). 15947456 10.1262/jrd.17001

[11] Peter, A. An update on cystic ovarian degeneration in cattle. *Reprod. Domest. Anim.* **39**, 1–7 (2004). 15129913 10.1046/j.0936-6768.2003.00466.x

[12] Jeengar, K. et al. Ovarian cysts in dairy cows: old and new concepts for definition, diagnosis and therapy. *Anim. Reprod. (AR)* **11**, 63–73 (2018).

[13] Silvia, W., Hatler, T. & Nugent, A. Da Fonseca, L. L. Ovarian follicular cysts in dairy cows: an abnormality in folliculogenesis. *Domest. Anim. Endocrinol.* **23**, 167–177 (2002). 12142235 10.1016/s0739-7240(02)00154-6

[14] Vanholder, T., Opsomer, G. & De Kruif, A. Aetiology and pathogenesis of cystic ovarian follicles in dairy cattle: a review. *Reprod. Nutr. Dev.* **46**, 105–119 (2006). 16597418 10.1051/rnd:2006003

[15] Perez, J., Conley, A., Dieter, J., Sanz-Ortega, J. & Lasley, B. Studies on the origin of ovarian interstitial tissue and the incidence of endometrial hyperplasia in domestic and feral cats. *Gen. Comp. Endocrinol.* **116**, 10–20 (1999). 10525357 10.1006/gcen.1999.7331

[16] Hillard, J., Spies, H. & Sawyer, C. Hormonal factoryes regulating ovarian choleslerole mobilization and progestin secretion in interstitial and hypo ghysectomized rabbits, appleton-century-crofts. *New. York Italiano Anat. Embriol.* **86**, 71–82 (1969).

[17] Tsvetkov, T., Stanchev, V. & Takeva, T. Comparative study of the ovarian interstitial gland in non-hibernating and hibernating mammals. (2003).

[18] Guraya, S. Histochemical observations on the civet cat (*Paradoxurus hermaphroditus*) ovary during the period of follicular activity. *Archivio Italiano Anat. Embriol. Italian J. Anat. Embryol.* **86**, 71–82 (1981). 7198430

[19] Hein, G. J. et al. Impaired insulin signaling pathway in ovarian follicles of cows with cystic ovarian disease. *Anim. Reprod. Sci.* **156**, 64–74 (2015). 25813700 10.1016/j.anireprosci.2015.02.010

[20] Gareis, N. C. et al. Impaired insulin signaling pathways affect ovarian steroidogenesis in cows with COD. *Anim. Reprod. Sci.* **192**, 298–312 (2018). 29622349 10.1016/j.anireprosci.2018.03.031

[21] Green, D. P., Limjunyawong, N., Gour, N., Pundir, P. & Dong, X. A mast-cell-specific receptor mediates neurogenic inflammation and pain. *Neuron* **101**, 412–420 (2019). 30686732 10.1016/j.neuron.2019.01.012 PMC6462816

[22] Goericke-Pesch, S., Schmidt, B., Failing, K. & Wehrend, A. Changes in the histomorphology of the canine cervix through the oestrous cycle. *Theriogenology* **74**, 1075–1081 (2010). 20580071 10.1016/j.theriogenology.2010.05.004

[23] Liu, Y., Yu, M., Wang, C., Peng, K. & Liu, H. Distribution patterns of mast cells in the uterus of pregnant Meishan pigs. *Reprod. Domest. Anim.* **47**, 574–577 (2012). 22066801 10.1111/j.1439-0531.2011.01920.x

[24] Asuman, Ö. et al. Light and electron microscopic studies on mast cell of the Sow oviduct. *Ankara Üniversitesi Veteriner Fakültesi Dergisi* **61**, 9–14 (2014).

[25] KÜRÜM, A., Karahan, Ö. Z. E. N. A. & ÖZCAN, Z. S. Investigation of mast cell distribution in the ovine oviduct during oestral and luteal phases of the oestrous cycles. *Kafkas Üniversitesi Veteriner Fakültesi Dergisi* **20** (2014).

[26] Karaca, T., Yörük, M., Uslu, S., Çetin, Y. & Uslu, B. A. Distribution of eosinophil granulocytes and mast cells in the reproductive tract of female goats in the preimplantation phase. *Vet. Res. Commun.* **33**, 545–554 (2009). 19184632 10.1007/s11259-009-9203-x

[27] Valle, G. et al. Eosinophils and mast cells in the oviduct of heifers under natural and superovulated estrous cycles. *Anim. Reprod. (AR)* **6**, 386–391 (2018). 10.1016/j.anireprosci.2006.08.026 17010540

[28] Schöniger, S. & Schoon, H. A. The healthy and diseased equine endometrium: A review of morphological features and molecular analyses. *Animals* **10**, 625 (2020). 32260515 10.3390/ani10040625 PMC7222714

[29] Walter, J., Klein, C. & Wehrend, A. Distribution of mast cells in vaginal, cervical and uterine tissue of non-pregnant mares: investigations on correlations with ovarian steroids. *Reprod. Domest. Anim.* **47**, e29–e31 (2012). 21950580 10.1111/j.1439-0531.2011.01897.x

[30] Komi, E. A., Shafaghat, D., Haidl, G. & F. & Significance of mast cells in spermatogenesis, implantation, pregnancy, and abortion: cross talk and molecular mechanisms. *Am. J. Reprod. Immunol.* **83**, e13228 (2020). 32053232 10.1111/aji.13228

[31] Campanile, G., Baruselli, P. S., Limone, A. & Michael, J. Local action of cytokines and immune cells in communication between the conceptus and uterus during the critical period of early embryo development, attachment and implantation–Implications for embryo survival in cattle: A review. *Theriogenology* **167**, 1–12 (2021). 33743503 10.1016/j.theriogenology.2021.02.020

[32] Nelson, S. T., Martin, A. D. & Østerås, O. Risk factors associated with cystic ovarian disease in Norwegian dairy cattle. *Acta Vet. Scand.* **52**, 1–10 (2010). 21059258 10.1186/1751-0147-52-60 PMC2990741

[33] Probo, M. et al. Reproductive performance of dairy cows with luteal or follicular ovarian cysts after treatment with Buserelin. *Anim. Reprod. Sci.* **127**, 135–139 (2011). 21920681 10.1016/j.anireprosci.2011.07.019

[34] Karaca, T., Yoeruek, M. & Uslu, S. Distribution and quantitative patterns of mast cells in ovary and uterus of rat. *Arch. Med. Vet.* **39** (2007).

[35] Bartolome, J. A., Thatcher, W. W., Melendez, P., Risco, C. A. & Archbald, L. F. Strategies for the diagnosis and treatment of ovarian cysts in dairy cattle. *J. Am. Vet. Med. Assoc.* **227**, 1409–1414 (2005). 16279384 10.2460/javma.2005.227.1409

[36] Douthwaite, R. & Dobson, H. Comparison of different methods of diagnosis of cystic ovarian disease in cattle and an assessment of its treatment with a progesterone-releasing intravaginai device. *Vet. Rec.* **147**, 355–359 (2000). 11083046 10.1136/vr.147.13.355

[37] Bottomley, C. & Bourne, T. Diagnosis and management of ovarian cyst accidents. *Best Pract. Res. Clin. Obstet. Gynecol.* **23**, 711–724 (2009). 10.1016/j.bpobgyn.2009.02.001 19299205

[38] Khaled, A., El-Nahass, E. S., Hussien, M., El-Nesr, A. & M. & Morphological pathology of bovine ovarian abnormalities in correlation to uterine changes. *J. Vet. Med. Res.* **23**, 191–198 (2016).

[39] Awad, M., Mohamed, R., Amin, Y. & Hussein, H. Histological and immunohistochemical investigations of ovarian interstitial glands during non-breeding season in camels (*Camelus dromedarius*). *Reprod. Domest. Anim.* **53**, 872–879 (2018). 29602226 10.1111/rda.13178

[40] Mohammed, A. H. S., Ali, T. J. & Naki, Z. J. Histological study of ovarian interstitial glands in IRAQI buffaloes at six and nine years old. *J. US China Med. Sci.* **10**, 170–173 (2013).

[41] Niimura, S. & Yokota, K. Postnatal development of interstitial glands in rabbit ovaries, with particular reference to the ability of steroidogenesis. (2001).

[42] Mori, H. & Matsumoto, K. Development of the secondary interstitial gland in the rabbit ovary. *J. Anat.* **116**, 417 (1973). 4208862 PMC1271576

[43] Deanesly, R. Origins and development of interstitial tissue in ovaries of rabbit and guinea-pig. *J. Anat.* **113**, 251 (1972). 4664236 PMC1271685

[44] Özen, A., Ergün, L., Ergün, E. & ŞİMŞEK, N. Morphological studies on ovarian mast cells in the cow. *Turkish J. Vet. Anim. Sci.* **31**, 131–136 (2007).

[45] Glavaski, M., Banovic, P. & Lalosevic, D. Number and distribution of mast cells in reproductive systems of gravid and non-gravid female mice. *Exp. Appl. Biomed. Res. (EABR)* **23**, 67–73 (2019).

[46] Hamouzova, P., Cizek, P., Jekl, V., Gozdziewska-Harlajczuk, K. & Kleckowska-Nawrot, J. Mast cells and Kurloff cells-their detection throughout the oestrous cycle in normal Guinea pig ovaries and in Guinea pigs with cystic rete ovarii. *Res. Vet. Sci.* **136**, 512–518 (2021). 33878613 10.1016/j.rvsc.2021.04.003

[47] Schmerse, F. et al. In vivo visualization of uterine mast cells by two-photon microscopy. *Reproduction* **147**, 781–788 (2014). 24534951 10.1530/REP-13-0570

[48] Meyer, N. & Zenclussen, A. C. Mast cells—good guys with a bad image? *Am. J. Reprod. Immunol.* **80**, e13002 (2018). 29917288 10.1111/aji.13002

[49] Hamouzova, P., Cizek, P., Novotny, R., Bartoskova, A. & Tichy, F. Distribution of mast cells in the feline ovary in various phases of the oestrous cycle. *Reprod. Domest. Anim.* **52**, 483–486 (2017). 28211113 10.1111/rda.12938

[50] Noor, N., Tripathi, T., Moin, S. & Faizy, A. F. Possible effect of histamine in physiology of female reproductive function: an updated review. *Biomed. Aspects Histamine Curr. Perspect.*, 395–405 (2011).

[51] Jensen, F. et al. Estradiol and progesterone regulate the migration of mast cells from the periphery to the uterus and induce their maturation and degranulation. *PloS ONE* **5**, e14409 (2010). 21203555 10.1371/journal.pone.0014409 PMC3008683

[52] Milne, S. A. et al. Co-localization of matrix metalloproteinase-1 and mast cell tryptase in the human uterus. *Mol. Hum. Reprod.* **7**, 559–565 (2001). 11385111 10.1093/molehr/7.6.559

[53] Szulańczyk, K. Histological changes within ovarian cortex, oviductal and uterine mucosa in case of ovarian cysts presence in sows. *Folia Histochem. Cytobiol.* **47**, 99–103 (2009). 19419946 10.2478/v10042-009-0005-3

[54] Villavicencio, A. et al. Androgen and estrogen receptors and co-regulators levels in endometria from patients with polycystic ovarian syndrome with and without endometrial hyperplasia. *Gynecol. Oncol.* **103**, 307–314 (2006). 16677694 10.1016/j.ygyno.2006.03.029

